# Ophthalmic equipment survey 2010: preliminary results

**Published:** 2010-09

**Authors:** Daksha Patel, Elizabeth Mercer, Ingrid Mason

**Affiliations:** MSc Course Director and Clinical Lecturer, International Centre for Eye Health (ICEH), London School of Hygiene and Tropical Medicine, Keppel Street, London WC1E 7HT, UK.; Courses Promotion and Scholarship Administrator, ICEH.; CBM Capacity Development Officer and Medical Advisor, PO Box 58004, 00200 City Square, Ring Road Parklands, Nairobi, Kenya.

**Figure:**
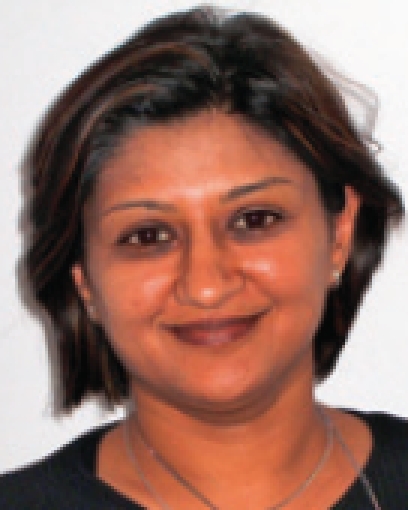


**Figure:**
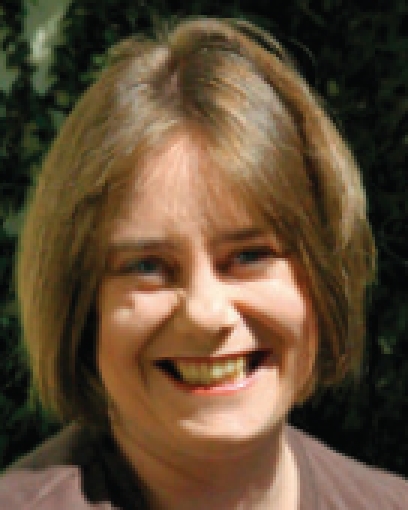


**Figure:**
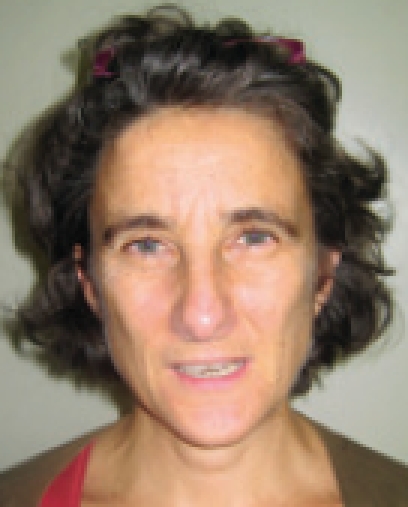


The delivery of ophthalmic services at all levels is completely dependent on equipment: from the simple torch light to the highly sophisticated equipment used for diagnosis and treatment.

In order to achieve the aims of VISON 2020: The Right to Sight and eliminate avoidable blindness by the year 2020, it is not enough to have the right equipment available at all levels of service delivery; there has to be a good maintenance and repair support service.^1^

The purpose of this equipment survey (commissioned by this journal) was to obtain an overview of the key issues and challenges faced by eye health providers with regard to their equipment.

The main objectives of the survey were:

- To identify what essential equipment was available and functional, based on the *IAPB Standard List of Equipment, Drugs and Consumables for a VISION 2020 Eye Care Service Unit* (Standard List),^2^ and where this equipment was
- To establish how much of the essential equipment was not working, the reasons equipment was not working, and for how long equipment remained that way
- To identify the impact on the provision of eye care services when equipment did not work.

## Survey methods

The Bristol Online Surveys tool was used to implement the questionnaire and to collect the data online. The questionnaire was based on the equipment in the Standard List and refined after pilot testing with the students enrolled in the International Centre for Eye Health (ICEH) Community Eye Health MSc course. The questionnaire required participants to give numerical responses and to share their comments and views.

The finalised questionnaire was circulated by email to members of the ICEH alumni network and the International Council of Ophthalmologists as well as to participants in the various VISION 2020 Links programmes. The survey was also made available on the ICEH website for visitors in charge of eye units to provide information. Data collection was active between 24 January and 24 April 2010. Only one questionnaire was completed per eye unit. This simple survey was not designed to obtain a representative sample across regions or countries, but rather to capture the key trends and themes with regards to equipment.

## About the participants

**Figure 1:**
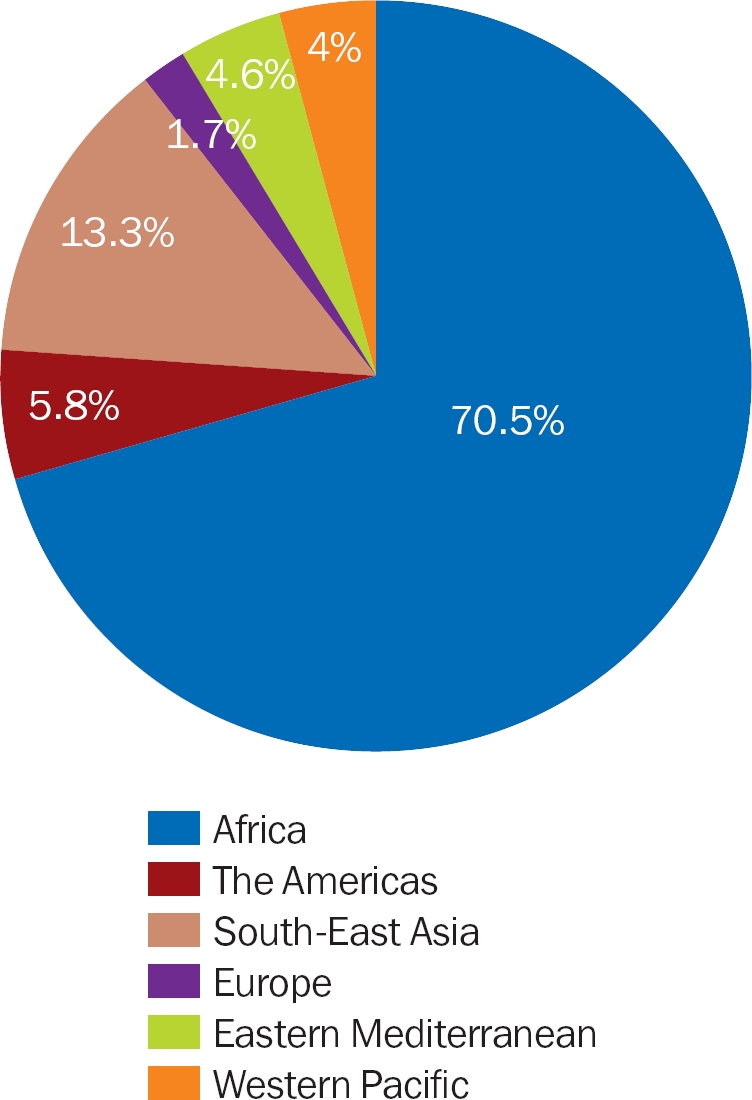
Regions represented in the survey

We received 173 responses, 55.7% of which were from training facilities (tertiary hospitals). Over two-thirds of the respondents were from Africa (Figure 1).

Background information on the main source of funding for each eye unit was also collected as this affects procurement of new equipment as well as maintenance and repair. Overall, half (50.9%) the responses were from government hospitals, 21.8% were from non-governmental organisations (NGOs) or mission hospital settings, whereas the remaining were either from private or insurance company-supported institutions. In Africa, 80% of all training institutions were government funded, compared to only 18% in South-East Asia.

Encouragingly, 71.1% of the eye units knew about the Standard List; this proportion was similar across the different regions.

## What equipment was available and working?

Overall, the private and NGO sectors were better equipped than government ophthalmic units.

This was true in all regions surveyed and across the full range of equipment covered in the survey.

### Cataract surgery

**Figure 2:**
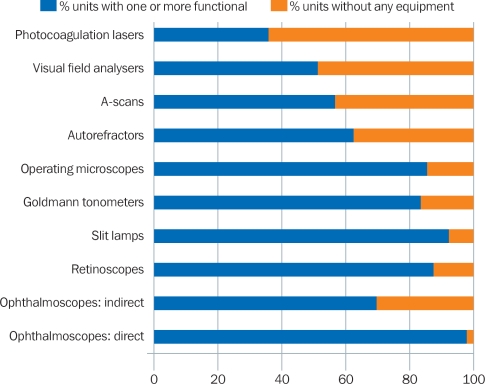
Availability of equipment

In total, 80% of the units reported that, of the equipment required to provide basic cataract surgery and follow up (operating microscope, slit lamp, ophthalmoscope, and retinoscope), they had at least one that was functional (Figure 2).

However, only 57% of all units had an A-scan for carrying out biometry. Without biometry, surgeons cannot select the most appropriate intraocular lens (IOL) power and patients may need optical correction after surgery. Within Africa, only 38% of the eye units reported having a functional A-scan.

When considering training institutions separately, we found that 79.4% of the training institutions in Africa reported having no working A-scans. This was still a problem in the Eastern Mediterranean and Western Pacific regions (50% had no A-scan), but less so in South-East Asia (just 11%).

If the results can be assumed to be representative of the different regions, it is cause for concern that so few cataract surgeons in Africa, the Eastern Mediterranean region, and the Western Pacific region have the opportunity to be trained in routine biometry.

### Refractive error

Refractive error diagnosis was possible at 87% of the units who responded, as they had at least one functional retinoscope. Over 63% even had an autorefractor.

### Glaucoma

Encouragingly, 78% of the units in Africa and 97% in South-East Asia reported having at least one tonometer. A total of 14% of units in Africa reported that non-functioning tonometers remained unrepaired for over a year, mainly as no one was trained to identify and manage the technical problems that occurred. Over half of the eye care institutions responding from Africa and South-East Asia had no visual field analysers; this highlights the need to strengthen quality glaucoma management in these regions.

## Equipment that had stopped working

We were interested to find out:

- In which eye units equipment had stopped working
- Why equipment had stopped working
- How long equipment didn't work for, and why.

### Where was the equipment?

**Figure 3:**
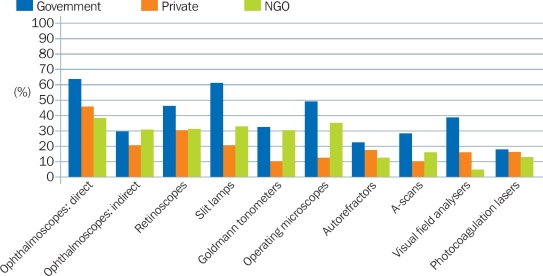
Percentage of eye units with one or more items of equipment that did not work (by provider)

Figure 3 highlights the challenges faced in the government sector (the main health service provider in many countries) compared to the NGO and private sectors. In all instances, more government eye units had equipment that did not work. Notably, 60% of the government units reported that one or more slit lamps did not work.

### Why did the equipment stop working?

The causes were divided into:

- Easily manageable causes: blown bulbs, faulty electrical connections, blown fuses, etc.
- Preventable causes: poor maintenance, inadequate cleaning, breakages during transport, etc.
- Unknown or complex technical causes.

**Figure 4:**
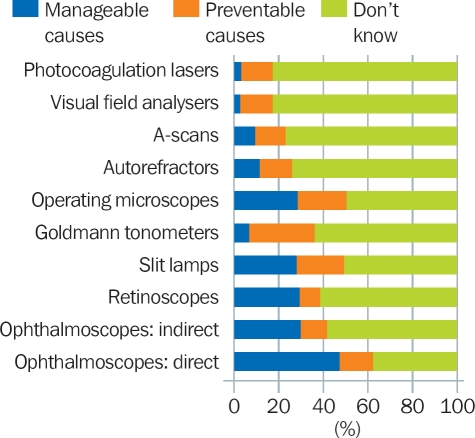
Causes of equipment not working

On average, easily manageable or preventable causes were responsible for more than a third of the equipment that had stopped working (Figure 4). Breakage due to poor handling, for example, “being dropped” or “damage during travel to outreach,” raised questions about the care taken with equipment.

### For how long did the equipment not work, and why?

**Figure 5:**
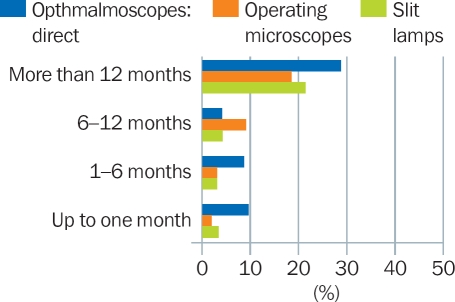
Percentage of eye units in which basic equipment did not work for different time periods

On average, over 20% of all the eye units who responded to the survey reported that they had equipment which was not working for more than 12 months (Figure 5). In one extreme case, slit lamps were not working for over 15 years.

**Figure 6:**
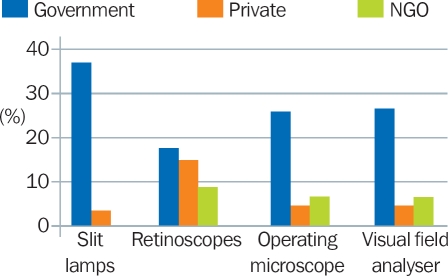
Percentage of eye units with equipment that was non-functioning for over a year, by provider

The key trend noted was that equipment not working for longer than a year was predominately a problem within government hospitals. For example, 59% of government units reported that slit lamps remained unrepaired for more than 12 months, compared to 3% in private settings and 0% in NGO settings (Figure 6).

### Why did equipment not work for long periods of time?

One of the common reasons that equipment did not work for long periods of time was that the model was too old and that spare parts were not available; this was true for slit lamps, retinoscopes, indirect and direct ophthalmoscopes, and visual field analysers in particular.

“No-one to fix it” was a common reason given in the African region.

Lack of funding, especially in government settings, was raised as a major barrier in all regions.

**Figure 7:**
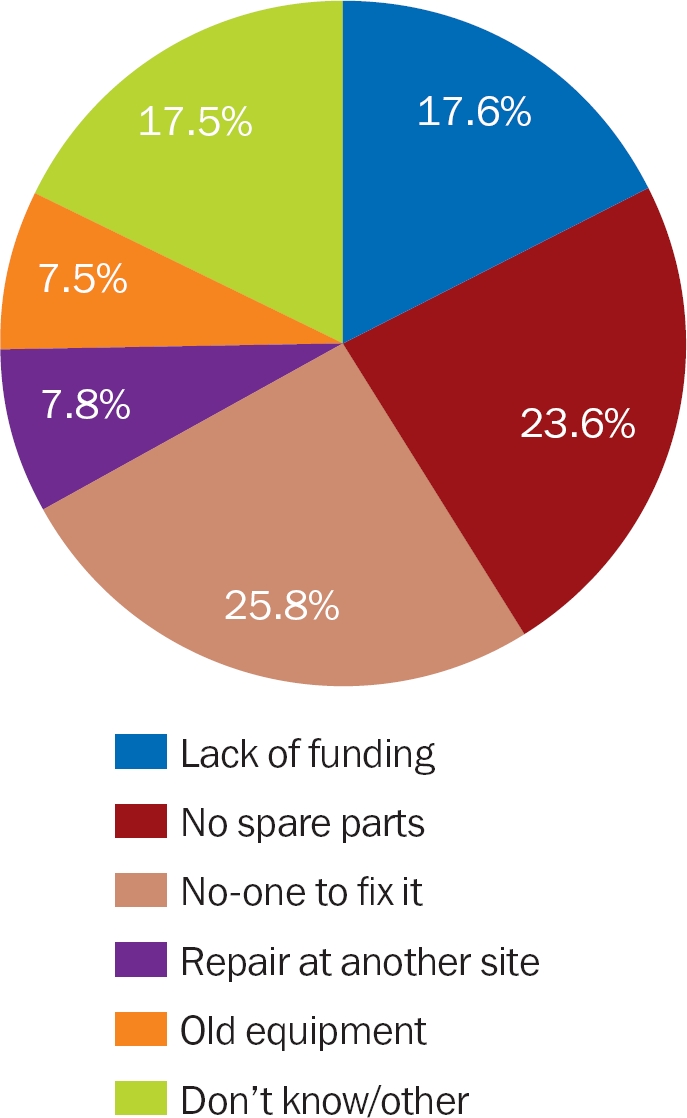
Reasons equipment did not work for a period of time

In total, 60% of the units indicated that they had no reporting system or log for faulty equipment. Furthermore, there was no designated person to take responsibility for the equipment that did not work. This could help to explain the delays in arranging for repairs.

Nursing staff in only 31% of the units had received any form of training to maintain or clean the equipment.

Specialist training for technicians was available for only 33% of the eye units overall. In total, 51% of the eye units reported having access to the services of a trained general technician. One of the respondents pointed out that access to a general technician was not sufficient: “We have two medical technicians who are looking after all the medical equipment in the hospital. We need somebody who can [teach] them ophthalmic instrument maintenance.”

**Figure 8:**
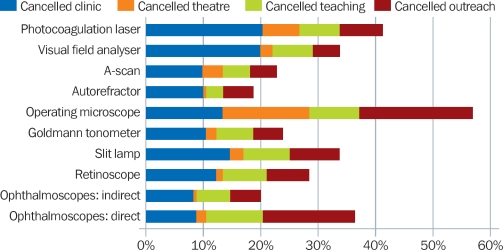
Percentage of eye units that cancelled services due to lack of functioning equipment

### The impact of equipment that did not work

Some eye units have had to cancel or reschedule clinics and operations when their equipment broke down. Outreach programmes in almost 20% of the eye units were cancelled at some point due to lack of operating microscopes, which meant that screened patients have had to be turned away.

For both outreach services and those at the clinic, the inconvenience to patients is great, particularly in rural areas where patients often have to travel long distances. Long-term or repeated cancellations result in disappointment and loss of trust. This can damage the reputation of the eye care service and will have an impact on its ability to attract patients in future.

The impact of breakdowns was described as “increased waiting times for patients”, “delays due to sharing of equipment”, and “referral without a proper examination.” In addition, inability to conduct a proper preoperative assessment (due to non-functioning slit lamps) increases the risk of complications and poor visual outcomes.

Delays and cancellations are frustrating for eye care staff and have an impact on their motivation; this will in turn diminish their ability to deliver high-volume, high-quality services. As a result, retention of trained professionals in poorly equipped centres may become a challenge.

## Problems with donated and surplus equipment

Donations reported by respondents included sophisticated diagnostic equipment which was not a priority requirement. These included equipment for fluorescein and indocyanine green (ICG) angiography, optical coherence tomography (OCT), and a Heidelberg Retina Tomograph (HRT).

Some donated equipment was not working because it required specific accessories that were either difficult to obtain or unaffordable. One of the respondents noted as follows: “[…] three donated virectors but only one is working. From the beginning they needed different accessories that must be bought in order to use the machine.”

Some donated equipment no-one knew how to use. Respondents also did not know why some items were given to them (in some cases, the items were purchased by central government suppliers). These items included lasers for retinal photocoagulation, phacoemulsification machines, and retinoscopes. In one instance, a respondent reported being unable to use donated retinoscopes “because their training is in [a] French system.”

Some equipment was donated “without warranty or instructions for use and handling,” as reported by an African respondent.

Other equipment, such as A-scans, ultrasound appliances, OCT, and Yag lasers, had been purchased but was awaiting assembly for a long period of time (over six months). There are several possible reasons: because the equipment was not really needed, because there was no-one assigned to take responsibility for it, or because there was no-one who was able to assemble it.

## Recommendations

- All clinical staff should be trained in basic maintenance of commonly used equipment for a district level eye unit.
- When new equipment is purchased, staff should be instructed in the basic care and maintenance that the equipment requires.
- Every unit must nominate an ‘equipment person’ who has a keen interest in maintaining equipment. This person should be supplied with a clear job description, which includes maintaining an inventory list for equipment and spare parts, reporting on the functionality of equipment, and tracking repair work. This person should have undergone at least some basic training in equipment maintenance.
- More ophthalmic and biomedical technicians need to be trained in ophthalmic equipment maintenance.
- A module on the maintenance and repair of commonly used equipment found at the district eye unit should be developed and embedded into the training curriculum of all mid-level eye care workers.
- Local or regional equipment maintenance and repair training centres should be established.
- Donors of equipment should inform the potential recipient what is being donated and what support (consumables, spare parts, maintenance, water and electrical supply) will be required. Before accepting the donation, the recipient must ensure that they can fully support the equipment and that they have the budget to do so (see article on page 32).
- New items of equipment should be purchased with all spare parts and consumables for at least the first year of use (see article on page 34).
- Arrangements need to be made ahead of time for the maintenance and repair of both donated and purchased equipment.
- Newly purchased equipment should be installed by the manufacturer or supplier, where possible, and training given to staff on the basic care and maintenance that the equipment requires.

Equipment is central to service delivery and quality and is closely linked with the motivation of eye care personnel to do their job. More efficient, effective, and long-term use of equipment will be possible if eye units are able to acquire appropriate equipment which meets their needs, which they are trained to use and care for, and which they can afford to maintain.
